# A Conceptual Map of Knowledge Transfer in Occupational Therapy Clinical Practice in Central South Africa

**DOI:** 10.1155/2024/8873026

**Published:** 2024-09-04

**Authors:** Azette Swanepoel, Corlia Janse van Vuuren, Shoba Nayar

**Affiliations:** ^1^ Department Occupational Therapy University of the Free State, CR de Wet Bophelong Building, Rectors Road, 205 Nelson Mandela Drive, Park West, Bloemfontein, South Africa; ^2^ School of Health and Rehabilitation Sciences University of the Free State, CR de Wet Bophelong Building, Rectors Road, 205 Nelson Mandela Drive, Park West, Bloemfontein, South Africa; ^3^ Independent Academic, India

## Abstract

**Introduction:** Empirical evidence has confirmed that all types of knowledge (propositional, procedural, personal, and client) contribute to evidence-based practice (EBP) and should be transferred in clinical practice to inform quality service delivery. However, it is unclear how the integration of the types of knowledge that are transferred in clinical practice manifests. Given this gap in understanding, the current research sought to build a conceptual map of knowledge transfer in clinical practice in central South Africa.

**Method:** A qualitative approach was followed, and data to build the conceptual map was obtained from a scoping review that explored the landscape of knowledge transfer in occupational therapy clinical practice, nine semistructured interviews with occupational therapists working in central South Africa, and a Q Method survey.

**Results:** The conceptual map–building process delivered a multidimensional, multidirectional conceptual map consisting of four concepts (theory and research, practice experience, patient–therapist relationship, and patient's voice in clinical practice) and four types of knowledge (propositional, procedural, personal, and client). The results show the integration of the types of knowledge and confirm that knowledge transfer in clinical practice is a complex and ongoing process.

**Conclusion:** The conceptual map, a first of its kind in South Africa, presents empirical evidence of knowledge that is created and transferred in clinical practice in central South Africa. The conceptual map might provide a framework for collaboration amongst all stakeholders, such as patients, occupational therapists, and academics, to produce practice guidelines and occupational outcome measures to support evidence-based clinical practice.

## 1. Introduction

Occupational therapists and academics agree that different types of knowledge inform clinical practice. A scoping review [1], by the first author, of the types of knowledge used in occupational therapy clinical practice, that is, propositional, procedural, personal, and client knowledge, was all found to be transferred in clinical practice during the occupational therapy process.

The topic of knowledge transfer has been widely debated, with authors often using the term interchangeable with knowledge translation. Several frameworks and models have been designed to inform clinical practice and provide research knowledge for clinical practice [2–4]. However, the practice–theory gap of research knowledge uptake in clinical practice remains a challenge, giving rise to the process of knowledge translation. Various definitions of knowledge translation exist; however, the definition formulated by the Canadian Institute for Health Research (CIHR) has been adopted for this study: Knowledge translation is “a dynamic and iterative process that includes synthesis, dissemination, exchange and ethically sound application of knowledge to improve the health of Canadians, provide effective health services and products and strengthen the healthcare system” [5]. This definition implies that knowledge translation is an entire process of identifying a research question, planning and implementing a study, and synthesising results, to dissemination or implementation of the research results in clinical practice. Multiple stakeholders, such as policymakers, funders, health service managers, clinicians, and researchers, are involved in the process to deliver evidence-based knowledge to clinical practice [3]. Further, the CIHR definition of knowledge translation implies a one-way process that has the aim to deliver research and facilitate utilization of research knowledge in clinical practice.

However, knowledge transfer, which is the focus of the current study, is seen as a subcategory in the knowledge translation process and happens in clinical practice. Graham et al. and Taylor, Fisher, and Kielhofner [3, 6] describe knowledge transfer as “a two-way process of getting knowledge used by stakeholders and usually encompasses all forms of knowing.” The content of knowledge that is transferred to clinical practice includes both the research evidence and practical experience of the occupational therapist, and client knowledge [3, 7, 8]. Knowledge, therefore, is not transferred *to* practice but rather transferred *in* practice between clinicians, patients, and other stakeholders. Moreover, knowledge is created when transferred in clinical practice between all stakeholders [3, 7]. It is obtaining an occupational history of a client or therapist sharing their experience to solve a clinical question. Because of its all-encompassing nature, knowledge transfer as a method of informing practice was the focus of this study.

Propositional (theoretical/empirical) knowledge is knowing that a concept is true and it should meet the conditions of justified belief [9]. This type of knowledge, also known as theoretical or scientific knowledge [10], is where a concept is constructed based on the belief that it represents truth; for example, meaningful occupational engagement improves health and well-being. The concept is scientifically justified and taken up in the body of knowledge of the profession. Propositional knowledge is therefore based on empirical evidence that justifies the theoretical constructs proposed as occupational therapy theory. This type of “knowing that” knowledge is seen as an important contributor to quality service delivery to patients in evidence-based practice (EBP) settings [11]. In EBP, the application of propositional knowledge is often reliant on the occupational therapist utilizing their theoretical knowledge when they consider empirical evidence. A therapist's knowledge of occupational therapy theory is contained in conceptual or practice models and frameworks and should inform their choice of empirical evidence to ensure that the best evidence is transferred in clinical practice. An attempt to follow an EBP approach in clinical practice in the absence of the necessary theoretical knowledge is futile because the occupational therapist would not be able to recognize the correct evidence to use in their specific clinical setting if they do not have the theoretical knowledge [12].

Because of propositional knowledge, therapists recognize the value of occupational engagement for a patient to restore or maintain well-being. The interaction between propositional and procedural knowledge during knowledge transfer in clinical practice is, however, complex. A wealth of procedural (clinical experience) knowledge is transferred in clinical practice when occupational therapists make use of their clinical experience to critically reason about the best outcomes for their patients [13, 14]. When the patient's context, for example, is not supported by empirical evidence in practice guidelines, therapists fall back on their clinical experience to deliver quality service to their patients [15]. Moreover, therapists develop skills to observe certain traits and behaviors of patients, read small nuances and body language [16], and understand contextual factors that might influence the therapeutic process of a patient. Through procedural knowledge, therapists acquire tacit skill sets and are able to plan contextually relevant assessments and interventions for each patient [7].

Clinical practice is also informed by the personal knowledge of the therapist. It appears that there has been limited focus on the transfer of personal knowledge in clinical practice. This type of knowledge is internal and contextually relevant to each therapist. The therapist's worldview, beliefs, values, and self-knowledge manifest in clinical practice and often develop through clinical experience and personal reflection [6, 10]. Personal knowledge, as is the case with the other types of knowledge, adds to the complexity of knowledge transfer in clinical practice. This person-specific knowledge held by each therapist has been shaped and influenced by contextual factors that might be a barrier in their clinical practice. For example, if a therapist is treating a patient whose behavior does not correlate with their values and belief systems, there might be tension in the patient–therapist relationship. Instead, if the clinician's belief is to always have the best interest of the patient at heart, they would not let their values and beliefs interfere in the treatment of the patient or interactions with caregivers.

Therapists gain information from patients and/or caregivers to inform their assessment and intervention practice. Client knowledge refers to the expert knowledge that a patient transfers in the therapeutic process. Patients have first-hand experience with their environments, performance skills and patterns, support structures, and occupational challenges [7, 16], and where the patient's pathology allows, they can share this knowledge with therapists. Let us use the intervention outcome of ADL, hygiene as an example. During an assessment, the patient or patient's caregiver will provide information regarding resources such as running water and electricity in their home, something that might be taken for granted by many therapists. The intervention will then include where, how, and by whom will water be collected for the patients to wash themselves. Patients might also share the structure of their homes which will further inform the intervention. If a patient's home does not have a bathroom, personal hygiene activity might take place in the kitchen or the patient's bedroom. By using this contextual information provided by the patient or their caregivers, the occupational therapist will be able to design an intervention plan specific to the occupational needs of the patient.

In a diverse healthcare setting such as South Africa, with its 12 official languages, various cultural practices, vast rural context, and often limited human and nonhuman resources [17], occupational therapy services utilize all types of knowledge to ensure contextually relevant service delivery. Yet, it is unclear, in the South African context, how (1) the content of the different types of knowledge is created and (2) an integration of the types of knowledge that are transferred in clinical practice would manifest. This article reports on the development of a conceptual map that depicts how knowledge might be created and how all the types of knowledge are transferred in occupational therapy clinical practice in central South Africa.

## 2. Methodology

Ethical approval for the study was received from the Health Science Research Ethics Committee (UFS-HSD2021/1454/2610) of the University of the Free State.

**Figure 1:**
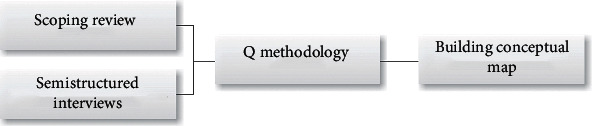
Methods to identify concepts of the conceptual map of knowledge transfer.

This study was part of a larger study that included a scoping review, semistructured interviews, and Q methodology (see Figure 1). To build a conceptual map of knowledge transfer in occupational therapy clinical practice in central South Africa, a qualitative approach was followed, utilizing the concept mapping method described by Canas and Novak [18]. Concept mapping was developed by Novak in 1972, based on Ausubel's assimilation theory [18–21], which suggests that meaningful learning is enhanced when (i) the researcher investigates ways to link newly obtained knowledge (e.g., obtained from this study) to existing knowledge, (ii) the researcher holds knowledge of the subject under investigation to be able to form new concepts, and (iii) the new knowledge and information are meaningful and applicable to, for instance, occupational therapy clinical practice. The theoretical assumption of knowledge in terms of conceptual maps is that knowledge is created by people, a notion that is supported by the social constructivist paradigm [18]. During the conceptual map-building process, the first author was actively involved in creating concepts to include in the conceptual map to gain an understanding of how an integration of the types of knowledge transferred in clinical practice would manifest. The first author acknowledges the social constructivist paradigm construct that the concepts of the conceptual map were created in a specific context at a specific time that might change over time. Conceptual maps represent subjectively constructed knowledge and contain, in the case of this study, empirical evidence [21]. A systematic process of building the conceptual map of knowledge transfer in occupational therapy clinical practice was followed based on the description by Canas and Novak [18]. The process was divided into four steps, as explained below.

### 2.1. Step 1: Formulate a Focused Question

Canas and Novak [18] suggested that focused questions should be formulated that will be answered by the conceptual map. The question that informed the development of the conceptual map included in this article was as follows: How are all the types of knowledge created and transferred in occupational therapy clinical practice in central South Africa?

### 2.2. Step 2: Identify Concepts to Be Included in the Map

This step of the conceptual map building was completed through three data generation methods as shown in Figure 1.

First, a scoping review was conducted to gain an understanding of the perceived importance of content and types of knowledge that are transferred in occupational therapy clinical practice globally. Second, semistructured interviews were conducted with nine occupational therapists from different practice settings in central South Africa to determine the content and types of knowledge that are transferred in occupational therapy clinical practice. Convenience sampling was used for this phase of the study. The thematically analyzed data of the scoping review and semistructured interviews informed the third method from which the concepts were identified, namely, Q methodology.

In Q methodology, the subjective perspectives of participants on a specific topic are determined [22]. For this study, Q methodology as described by Webler, Danielson, and Tuler [23] was followed using online QMethod Software [24]. The method required 14 participants to rank 42 statements from most applicable to least applicable. The statements pertained to the content and types of knowledge (propositional, procedural, personal, and client knowledge) transferred in clinical practice and were obtained from the scoping review and the semistructured interviews. Factor analysis of the ranked statements yielded two factors. A subsequent thematic analysis of statements that contributed to the two factors yielded four themes (henceforth referred to as concepts) that were used in Step 3, building the conceptual map. The four concepts of knowledge transfer in clinical practice are theory and research, practice experience, patient–therapist relationship, and patient's voice in clinical practice (see Table 1). During the thematic analysis process, a correlation between the four concepts and types of knowledge was identified.

### 2.3. Step 3: Moving Concepts Into the Map

**Figure 2:**
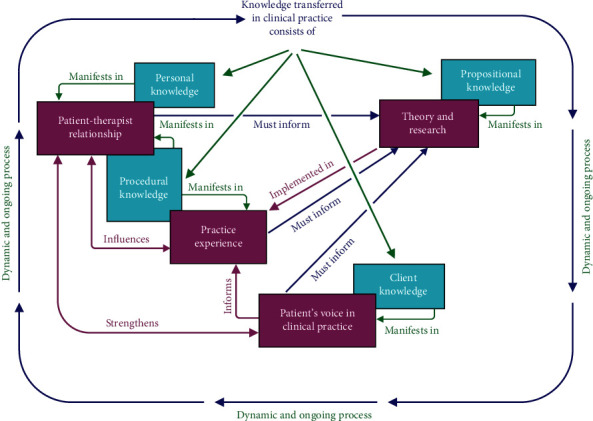
Conceptual map of knowledge transfer.

The four concepts identified during the Q methodology, and four types of knowledge, were moved to the conceptual map and placed in text boxes. This third step recommends that arrows or lines are used to indicate the link between concepts. Linking words, usually verbs, were placed on the arrows to form propositions that present the relationship between the concepts (see Figure 2). Canas and Novak [18] recommend hierarchical placement of concepts on the map, with concepts of more importance placed at the top of the conceptual map. This posed a challenge in the study, because all the types of knowledge transferred in clinical practice were of equal importance. Through respondent validation (see Step 4) and an iterative refining process by the authors, the placement of concepts in the map was done in a way that depicts the relationship between the different concepts best.

### 2.4. Step 4: Revision of the Conceptual Map

The conceptual map was revised by utilizing a respondent validation method and input from the study supervisors. Respondent validation was conducted with the nine participants who took part in the semistructured interviews. The participants already had a baseline understanding of the four types of knowledge, which had been explained to them during the semistructured interviews. A first draft of the conceptual map and a table (see Table 2) containing the concepts, categories of each concept, and examples were sent to participants for their comments. Five participants provided feedback on the first draft, and all agreed on the concepts and the categories of each concept. Four participants suggested editorial changes to the structure of the conceptual map. Two participants suggested additional content for the categories. Feedback was included in the second draft conceptual map of knowledge transfer in occupational therapy practice in central South Africa, which was then reviewed with the second and third authors. After an iterative refining process that involved cross-linking concepts to demonstrate the complex and interdependency of the different types of knowledge transferred in clinical practice, a graphic designer was approached to render a final electronic version of the conceptual map of knowledge transfer.

### 2.5. Trustworthiness of the Study

The credibility of the study was ensured by prolonged engagement with the topic, and by the methods utilized, triangulation. Method and data triangulation were used to obtain different data sets through the different methods that informed the conceptual map-building process. Theory triangulation was done through the inclusion of qualitative statements from the scoping review as a basis for the quantitative analyses in the Q methodology. Transferability and dependability were promoted by providing a thick description of knowledge transfer in clinical practice and by maintaining an audit trail of all documentation, data, and notes [25].

## 3. Results

The steps to build the conceptual map described above delivered a multidimensional and multidirectional conceptual map consisting of eight concepts (four themes and four types of knowledge), as presented in Figure 2 and Table 1. The conceptual map (see Figure 2) depicts knowledge transfer in occupational therapy clinical practice that consists of the four types of knowledge, namely, propositional, procedural, personal, and client knowledge. The correlation between the four concepts and types of knowledge is depicted, multidimensionally, by overlapping text boxes:

• Propositional knowledge correlates with and manifests in theory and research transferred in clinical practice.
• Procedural knowledge correlates with and manifests in practice experience and the patient–therapist relationship in clinical practice.
• Personal knowledge correlates with and manifests in the patient–therapist relationship.
• Client knowledge correlates with and manifests in the patient's voice in clinical practice.

The interrelated nature of the conceptual map will be discussed with reference to the four concepts and the four types of knowledge. Since knowledge transfer in clinical practice is an ongoing process, with no hierarchical flow, the following presentation of the findings will illustrate how knowledge transfer happens in an integrated manner (see Figure 2). The qualitative evidence was provided by participants during the semistructured interviews.

### 3.1. Patient's Voice in Clinical Practice

Patients enter the therapy process and are often able to transfer expert knowledge of their environment, occupational profile, occupational challenges, and support systems. Participants reported that the patients' knowledge of their socioeconomic environment guides the assessment and treatment approaches. South Africa has an unemployment rate of 32.9% [26], which might indicate that a large proportion of patients who are managed in government healthcare settings come from low socioeconomic contexts. Therapists consider the environment to which the patients will return using knowledge of the physical and sociopolitical context that is provided by the patients.

However, it might not only be the patient who engages in the transfer of knowledge during the therapeutic process. In many South African cultures, collaboration between the patient, therapist, and family members will determine the treatment or care that the patient should receive. Moreover, the intimate cultural knowledge that patients transfer in clinical practice gives patients a voice in the therapeutic process, as observed by Participant 2: “Each patient will come with their own values and beliefs, and each person's cultural/community context will add to how the knowledge is transferred and perceived.” Participants in the study agreed that the occupational therapist learns more from a patient's culture by engaging with and building relationships with patients. This relationship involves therapists listening to the patients, giving the patients a voice, and, in doing so, learning more about the finer nuances of a culture. Participant 8 commented that “Irrespective of the qualifications that we have, when you're in clinical practice, the stories the patients tell you really shapes you as a therapist, it also lets you look at theory in a different.” The patient's voice in clinical practice has the potential to strengthen the patient–therapist relationship. If one considers the valuable knowledge that is created in clinical practice, it is important that the patient's voice should inform the theory and research (propositional knowledge) of the profession.

South Africa has a rich cultural diversity; its people speak 12 official languages and have multiple cultural practices. However, the graduates of the eight occupational therapy training institutions in South Africa might be exposed only to the cultures that are predominant in the geographical area of the institution's training platforms. For contextually relevant knowledge to inform clinical practice, therapists, patients, and researchers should collaborate on how culture informs clinical practice and make the knowledge available to the profession.

### 3.2. Practice Experience

Theory (propositional knowledge) that is taught during the therapist's initial and postgraduate training is implemented as a foundation in clinical practice. Nevertheless, a wealth of practice experience (procedural knowledge) is continuously created in clinical practice by therapists and patients. The tacit knowledge therapists gain, which is often difficult to describe, was explained by participants as their intuition or “gut feeling” that guides their clinical practice. The more experience therapists have, the less they consciously fall back on their theoretical (propositional) knowledge because it has become engrained in their clinical practice experience.

Competencies that are mostly developed through clinical experience include listening and communication skills, ethical awareness, and empathy. Participant 7 said that empathy for her patients' circumstances developed through her practice experience: “experience that, I think creates a lot of empathy and a bit more understanding of what our clients go through and experience when we expect relatively simple things from them, which could actually be quite hard.”

Furthermore, the knowledge that patients transfer in clinical practice informs the occupational therapy process and, subsequently, therapists' practice experience. What therapists learn from one patient is often transferred to another patient with similar occupational challenges or cultural practices. Their approach to a particular patient might then be adjusted, because of the experience gained from another patient. Participants reported that their clinical reasoning developed through their clinical experience, and it frequently guided the consideration of research evidence (propositional knowledge) that informed their therapy. They can determine the relevance of the evidence in relation to a patient's pathology, occupational profile, socioeconomic context, and culture before the evidence is transferred in their clinical practice.

It is also in the clinical practice setting that therapists regularly reflect on their therapeutic processes. Knowledge is transferred between colleagues when they reflect on the effectiveness of interventions, collaborate to solve problems, and adjust their therapy accordingly because of such reflective practice.

### 3.3. Patient–Therapist Relationship

In this concept, occupational therapists' personal knowledge and practice experience (propositional knowledge) manifest in the patient–therapist relationship. The professional and ethical conduct of therapists in their clinical practice was demonstrated by participants who commented that they always put their patients first and strive to avoid being judgemental of their patients. In addition, it was reported that therapists do not let their personal values and beliefs interfere with or influence their relationship with patients: “You should not allow your own world views and values to influence your clinical reasoning or the way you treat your patient or clients” (Participant 9).

In practice settings where patients' pathologies allow, collaborative problem-solving between the patient and the therapist often takes place, with subsequent goal setting that aligns with the patient's occupational needs. In cases where the patient's pathology or age does not allow for collaboration with the therapist, the collaboration between caregivers and therapists results in person-centred care of, and service delivery to, patients.

The patient's voice in clinical practice informs practice experience, which, in turn, influences the patient–therapist relationship. The interplay between the patient and the therapist holds mutual benefits for the transfer of knowledge in clinical practice, in that the patient–therapist relationship is strengthened when the patient's occupational needs are met, because the therapist used, amongst others, the knowledge provided by the patient. The patient's voice is subsequently strengthened, because the therapist understands and utilizes the knowledge presented by the patient: “I think one of the ways that one gets the knowledge is actually through building relationships with the patient and having a lot of conversations” (Participant 2).

The patient–therapist relationship is sometimes challenged when, for example, language poses a barrier in the patient–therapist communication. The conversations mentioned by Participant 2 above are subsequently compromised because the therapist and the patient do not understand each other. Participant 3, who worked in a school for children with severe intellectual disabilities, explained that, because the children and the therapists do not always understand each other, the therapists use their theoretical knowledge, clinical experience, and observational skills during the therapy process: “We evaluate the children by observing them; how do they participate? And according to those behaviors we determine, for example, on which level of creative ability they function” (Participant 3). Participant 5 pointed to an exception of the rule and mentioned how, in her clinical practice setting, the patient–therapist relationship is negatively affected by the pathologies of her patients: “The dynamics of knowledge transfer is influenced when a patient presents with psychotic symptoms and does not have the relevant cognitive skills needed to participate in the process of knowledge transfer.” It might then be that the practice experience and theory and research are the primary aspects of the knowledge transfer process.

### 3.4. Theory and Research

Theoretical (propositional) knowledge is the foundation on which therapists base their clinical practice, and it manifests in the theory and research concept. Theory and research are transferred to practice through the tacit knowledge that therapists hold of an in-depth theory knowledge base. Participant 7 stated that “I've learned throughout my years of clinical practice it's obvious one needs a very strong theoretical foundation to be able to adapt and make decisions in your clinical reasoning.”

A code that none of the participants mentioned, but which is an important part of clinical practice, is policies and legislation that inform clinical practice (see Table 1). The reason for this omission might be that the transfer of policies and legislation in clinical practice has also become part of the tacit knowledge of participants, because it is considered daily with all patients.

## 4. Discussion

According to the social constructivist paradigm [27, 28] that underpins this study, knowledge can be created in clinical practice by occupational therapists, patients, members of the multidisciplinary team, caregivers and/or family members, and academics. Marques et al. [29] agree that knowledge can be created in clinical practice by all role players and argue that the creation of knowledge is important for the growth of any profession. The conceptual map provides a unique view of knowledge transfer in clinical practice and depicts the integration of all the types of knowledge utilized in clinical practice.

There are different views about how knowledge is created in healthcare. Graham et al. [3] present a knowledge-to-action (KTA) framework that depicts the knowledge translation process (of which knowledge transfer is a subcategory). According to the KTA framework, knowledge creation involves all types of knowledge and is depicted in three generations that have a close correlation with the concepts of the conceptual map (see Figure 2).

First-generation knowledge is presented as raw data or information that has not been analyzed, such as patient records or the occupational profile of patients or a community. The quality of the knowledge sources varies in numbers and quality. If one refers to the conceptual map, it becomes apparent that the patient's voice in clinical practice, the practice experience of therapists, and the patient–therapist relationship would be first-generation knowledge that holds potential knowledge that is yet to be processed or analyzed. The knowledge can be created by, for example, asking pertinent practice questions that can be answered by therapists and/or patients, or therapists writing up case studies that are shared with colleagues. Davis and Polatajko [7] stated that patients are experts of their occupational roles, role expectations, and occupational challenges; and the authors encourage therapists and academics to utilize this type of knowledge. Occupational roles in this study refer to, for example, fulfilling the role of a mother and caregiver of a child with cerebral palsy. The therapist might know, from literature, what challenges a child with cerebral palsy faces, but the expert knowledge of the mother will tell the therapist exactly what they are struggling with at home. The mother will be able to give a detailed account of how the diagnosis of the child impacts their home life, social engagements, and even the child's educational activities. She would also be able to offer insights into her culture's beliefs about illness and cultural practices to remedy illness or disease. However, this study found little evidence to show the value of understanding the patient's culture obtained directly from the patient to inform clinical practice. Moreover, propositional knowledge has a strong medical model focus; therefore, it remains the occupational therapist and academic's responsibility to ensure that occupation remains the focus of the occupational therapy process. This was demonstrated by Villa-Berges et al. [30], who reported, in a systematic review, that some studies on the efficacy of motor imagery for stroke patients did not consider the functional outcome of patients and only reported on the body functions that improved.

The second-generation knowledge creation in the KTA framework consists of the analysis and integration of existing knowledge. At this stage, knowledge is created by following predetermined methods to aggregate information linked to a practice question. Knowledge creation might take place in clinical practice, where therapists and/or researchers collaborate to find answers to a specific practice question. It might also happen during systematic reviews that follow a predetermined method. Therapists and/or researchers will evaluate all possible information from various data sources and apply the applicable knowledge in their clinical practice. This generation of knowledge creation would consist of the analyzed knowledge of the patient's voice and the practice experience of the therapist which strengthens the patient–therapist relationship. Haag et al. [31] demonstrated how, through the practice experience of therapists, their knowledge might be valuable in educating and/or preventing serious conditions, such as traumatic brain injuries of intimate partner violence survivors.

The third-generation knowledge creation suggests the production of “knowledge tools and products” [3] to inform and guide clinical practice. This is an important stage of knowledge creation for the occupational therapy profession that requires collaboration between colleagues, patients, caregivers, and other stakeholders to document the different types of knowledge that are transferred in clinical practice as presented by the conceptual map. Urquhart et al. [32] supported the notion that knowledge is created by colleagues who collaborate to produce empirical evidence that is disseminated in journal articles and conferences. Lo Bianco et al. [33] explained the value of collaboration between the occupational therapist and patients and highlight the subsequent negative outcome for patients if collaboration does not take place.

As depicted in the conceptual map, knowledge transfer is a multidimensional, dynamic, and ongoing process; thus, the creation of knowledge is dynamic and ongoing. The conceptual map of knowledge transfer in clinical practice can be used as a point of reference for therapists and academics to engage in collaborative knowledge creation.

### 4.1. Limitation

The small sample might have limited the feedback on how the different theories are transferred in clinical practice. Because of the small sample, the findings cannot be generalised to all South African occupational therapy clinical practice settings. Similar studies in other regions of the country might inform the transfer of knowledge in the different practice settings further. This study only involved occupational therapists in central South Africa, and it is recommended that a focused investigation is undertaken into patients' cultural practices and how they are transferred in clinical practice from the patient's perspective.

## 5. Conclusion

This paper set out to report on the development of a conceptual map that depicts how knowledge might be created and how an integration of the types of knowledge that are transferred in clinical practice would manifest in occupational therapy clinical practice in central South Africa. The conceptual map implies that knowledge transfer in clinical practice results from an integration of different types of knowledge: procedural, propositional, personal, and client. The process of building the conceptual map confirmed that knowledge is created in clinical practice by multiple role players, including therapists, patients, and academics. The process of knowledge transfer is continuous and dynamic, because no two occupational therapists have the same theoretical (propositional) knowledge, practice experience (procedural knowledge), or personal knowledge. Moreover, no two patients present similar signs and symptoms of a condition or disease, nor do they have similar values, beliefs, and contexts. Clinicians and academics need to share the wealth of their knowledge and, in doing so, build on the body of knowledge of the profession. Lastly, the conceptual map presented in this paper is a first of its kind in South Africa. Globally, conceptual maps have been used as teaching tools, yet no evidence could be found of a conceptual map explaining knowledge transfer in occupational therapy clinical practice.

## Figures and Tables

**Table 1:** Q methodology statements.

**Statement no.**	**Statement**	**Z** **-score**	**Sort values**	**Themes**
Factor 1 scores				
15	I put my patients first, I have their best interest at heart	2.14504	4	Patient–therapist relationship
26	Collaboration between my patients and me, through sharing of experiences and reflective practices, facilitates knowledge transfer that improves my service delivery	1.7739	4	Patient–therapist relationship
25	I demonstrate different skills to my patients or simulate the patients' environment	1.54671	3	Patient–therapist relationship
1	I use borrowed theories such as NDT, Behavioural, Cognitive behavioural, Client-centred, or Cognitive perceptual theoretic frames of reference, ICF, and Gestalt therapy in my clinical practice	1.30989	3	Theory and research
6	I use my experience, gut feeling and intuition to guide and adapt my therapy	1.17331	3	Practice experience
13	I use a combination of practical experience and theory knowledge in my clinical practice	1.09216	3	Practice experience
27	I listen to my patients; I give my patients a voice	0.92036	2	Patient–therapist relationship
21	My colleagues and I reflect, hypothesise and solve problems together, we share new ideas with each other	0.89184	2	Practice experience
18	Patients provide information such as their background, occupational profiles, role expectations, support systems, and home environment that I use in my clinical practice	0.81915	2	Patient's voice in clinical practice
28	My experience has taught me to use different approaches with patients, depending on their cultural practices, values, and beliefs	0.7287	2	Practice experience
23	I am authentic in my clinical practice and share my own stories and examples with my patients	0.68209	2	Patient–therapist relationship
35	I do not let my personal values and beliefs influence my clinical practice and relationship with my patients	0.59685	1	Patient–therapist relationship
32	I keep my patients' information confidential	0.58476	1	Patient–therapist relationship
16	I apply the theory of activity analysis in my clinical practice	0.54135	1	Theory and research
2	I use real life examples provided by my patients in my clinical practice	0.47246	1	Patient–therapist relationship
30	My clinical practice is based on research evidence to ensure quality service delivery to my patients	0.42869	1	Theory and research
34	I use observation as an evaluation method a lot	0.37823	1	Practice experience

Factor 2 scores				
13	I use a combination of practical experience and theory knowledge in my clinical practice	1.99126	4	Practice experience
21	My colleagues and I reflect, hypothesise and solve problems together, we share new ideas with each other	1.89884	4	Practice experience
24	The patients' pathology influences the choice of theory I use in my clinical practice	1.64481	3	Patient–therapist relationship
15	I put my patients first, I have their best interest at heart	1.58007	3	Patient–therapist relationship
38	My clinical reasoning is influenced by the knowledge I gain in my clinical practice	1.34444	3	Practice experience
31	I use patient feedback to establish if I am actually addressing the problem faced by the patient	1.12461	3	Patient's voice in clinical practice
28	My experience has taught me to use different approaches with patients depending on their cultural practices, values, and beliefs	1.12063	2	Practice experience
40	I use the knowledge that I have gained from negative experiences in my clinical practice	0.94771	2	Practice experience
6	I use my experience, gut feeling and intuition to guide and adapt my therapy	0.9336	2	Practice experience
34	I use observation as an evaluation method a lot	0.82764	2	Practice experience
10	My patients teach me more about their culture, values, and beliefs than what I can learn from theory	0.69287	2	Patient's voice in clinical practice
20	The patients' physical and socio-economic environments influence the choice of theory I use in my clinical practice	0.68237	1	Patient's voice in clinical practice
14	Patients contribute to the problem-solving process and their therapy	0.67311	1	Patient's voice in clinical practice
27	I listen to my patients; I give my patients a voice	0.6612	1	Patient–therapist relationship
32	I keep my patients' information confidential	0.58043	1	Patient–therapist relationship
36	I apply the knowledge that I gained from one patient to the next patient who has similar problems	0.44925	1	Practice experience
7	The patients' cultures influence the transfer of knowledge between me and my patients	0.14067	1	Patient–therapist relationship

**Table 2:** Concepts and categories of conceptual map.

**Concept**	**Category**	**Example**
Theory and research	Research evidence to ensure quality practice	Therapists read up on new research evidence, which is considered through clinical reasoning to evaluate the appropriateness of the evidence. Therapists hypothesise, look up research evidence, collaborate with colleagues, and/or apply the new knowledge to determine if their hypothesis is correct.
Propositional knowledge	Theory of activity analysis	Therapists report the use of activity analysis in their clinical practice. It often happens naturally after years of experience.
	Borrowed theory	NDT, ICF, and different theoretical frames of references are used in clinical practice.
	Policies and legislation	Missing code that informs clinical practice that was not obtained through interviews but stated in literature.
Practice experience	Combination of theory and experience	Theory is always the place to start; however, in some cases, actions in practice are based more on experience.
Procedural knowledge	Reflective practice with colleagues	Reflection with colleagues on difficult cases to determine what is best for a patient/client and discuss activities and/or techniques that work or do not work for a specific patient.
	Negative experiences guide current practice	Therapist will change treatment activities, approaches, and/or techniques because of negative clinical experiences with previous patients/caregivers/family members.
	Clinical reasoning through experience	Therapists use their experience to realize that each patient's context is different, which influences their clinical reasoning. When deciding on the use of different types of programs and different theoretical frames of references, therapists apply clinical reasoning based on the patient's age, pathologies, occupational profile, and context.
	Use of observation	Through experience and observations, therapists can conduct evaluations of patients.
	Experience, gut feeling, and intuition	Therapists use their experience to guide processes, such as the types of questions asked regarding patients' context at home and socioeconomic environments. Through experience, therapists know the appropriate processes to follow appropriate referral pathways. By doing regular assessments, therapists understand what to consider with each patient. Therapists are often set in their ways; they mostly use their experience in practice.
	Experience with different cultural preferences and values	Therapists know from experience how patients from different cultures experience their pathologies and how to engage with patients based on their culture.
Patient–therapist relationship	Put patients first	Therapists have patients' best interests at heart. Therapists update their knowledge and skills to ensure that patients receive the best treatment.
Procedural knowledge	Listen to patients; to give patients a voice	Therapists are the voice of vulnerable populations. Therapists give patients a voice because they look at patients holistically.
Personal knowledge	Demonstrate skills to patients	Therapists experience a skill or activity first before demonstrating it to a patient.
	Collaborate with patients	Therapists reflect with patients and review treatment progression; is therapist focusing on the relevant issues that are important to the patient? Therapists and patients collaborate to determine the main issues that need to be addressed in therapy. Therapists keep patients/clients up to date regarding the direction of therapy. Influenced by the pathology of the patients. If a patient's pathology prevents them from collaborating, therapists need to make intervention decisions to the benefit of the patient, often with input by caregivers—person-centred care.
	Use real-life examples	Giving patients real-life examples to explain the theory and skills that are presented to patients.
	Patient's occupational profile influences practice	Therapists gain knowledge regarding patients' occupational profile from and about the patients by building a therapeutic relationship with them.
	Patient's culture influences therapeutic relationship	Patients' culture influences the transfer of knowledge between the therapist and patient.
	Authentic therapists	Therapists share relevant life stories with patients in group sessions.
	Unbiased therapists	Therapists do not let personal values and beliefs influence clinical practice.
Patient's voice in clinical practice	Patient's occupational profile	Transfer what is learnt from one patient to another patient with similar situation/problems/pathology.
Client knowledge	Patient feedback	Patients provide feedback on therapy; therapists can determine if they are providing the service that the patient needs. This is a way to improve clinical practice and service delivery.
	Contribute to problem-solving	Patients provide information on their treatment needs and collaborate with the therapists when planning intervention strategies. Patients know their context the best and could come up with solutions if guided by the therapists.
	Culture and values	Patients teach therapists about their cultural practices and values. Patients learn from each other in treatment sessions and ask each other questions.
	Socioeconomic environment	Patients' socioeconomic environment has a definite influence on their therapy; it is considered during clinical reasoning by the therapists.
	Patient pathology	The patient's pathology is always considered in the clinical reasoning process that informs assessment and interventions.

Abbreviations: ICF: International Classification of Function; NDT: neurodevelopment therapy.

## Data Availability

All data are available and can be requested from the corresponding author.
